# Aerobic exercise improves motor dysfunction in Parkinson's model mice via differential regulation of striatal medium spiny neuron

**DOI:** 10.1038/s41598-024-63045-4

**Published:** 2024-05-27

**Authors:** Yinhao Wang, Longwei Wei, Mingli Tan, Zizheng Yang, Bo Gao, Juan Li, Yang Liu, Talifu Zikereya, Kaixuan Shi, Wei Chen

**Affiliations:** 1 School of Physical Education, Hebei Normal University, Shijiazhuang, China; 2 Key Laboratory of Measurement and Evaluation in Exercise Bioinformation of Hebei Province, Shijiazhuang, China; 3 Department of Physical Education, China University of Geoscience, Beijing, China

**Keywords:** Parkinson's disease, Motor dysfunctions, Aerobic exercise, Striatum, D2-MSNs, Neuroscience, Diseases of the nervous system, Motor control

## Abstract

The striatum plays a crucial role in providing input to the basal ganglia circuit and is implicated in the pathological process of Parkinson’s disease (PD). Disruption of the dynamic equilibrium in the basal ganglia loop can be attributed to the abnormal functioning of the medium spiny neurons (MSNs) within the striatum, potentially acting as a trigger for PD. Exercise has been shown to mitigate striatal neuronal dysfunction through neuroprotective and neurorestorative effects and to improve behavioral deficits in PD model mice. In addition, this effect is offset by the activation of MSNs expressing dopamine D2 receptors (D2-MSNs). In the current study, we investigated the underlying neurobiological mechanisms of this effect. Our findings indicated that exercise reduces the power spectral density of the beta-band in the striatum and decreases the overall firing frequency of MSNs, particularly in the case of striatal D2-MSNs. These observations were consistent with the results of molecular biology experiments, which revealed that aerobic training specifically enhanced the expression of striatal dopamine D2 receptors (D_2_R). Taken together, our results suggest that aerobic training aimed at upregulating striatal D_2_R expression to inhibit the functional activity of D2-MSNs represents a potential therapeutic strategy for the amelioration of motor dysfunction in PD.

## Introduction

Aging of the global population is a prominent demographic trend that has been accompanied by a rapid increase in the prevalence of neurodegenerative conditions such as Alzheimer's disease (AD), Parkinson’s disease (PD), and mild cognitive impairment^1,2^. Among these, PD is one of the common prevalent neurodegenerative conditions, with a significantly higher incidence among individuals aged > 60 years^3^. The underlying pathology of PD involves the progressive degeneration of dopamine (DA)-producing neurons, primarily in the substantia nigra pars compacta (SNpc). This neuronal loss leads to a substantial reduction in striatal DA levels, resulting in widespread motor and nonmotor dysfunction^4^.

The basal ganglia, a collection of subcortical nuclei, coordinates autonomous motor behaviors in the human body. In PD, the lack of DA in the striatum disrupts the internal motor control loop within the basal ganglia, thereby contributing to significant motor impairment^5^. The conventional model posits that the basal ganglia comprise two parallel neural circuits (direct and indirect pathways) originating from distinct subtypes of medium spiny neurons (MSNs) in the striatum^6^. In general, these two pathways exert opposing effects on the regulation of autonomous movement. The direct pathway acts as a "go" signal to facilitate motor behavior, while the indirect pathway serves as a "no-go" signal to inhibit motor behavior^7^. These DA-driven pathways can be activated concurrently during specific motor behaviors, suggesting a collaborative role in regulating autonomous movement^8,9^. Both Spike and local field potentials (LFP) are fundamental components of brain activity. LFP oscillations are the sum of local membrane currents and synaptic activity in the brain, reflecting afferent activity between neurons. Abnormal LFP patterns are considered to be key biomarkers of brain dysfunction. Changes in synchronised activity between multiple nuclei on the PD basal ganglia loop, particularly in the beta band of LFP^10^. Furthermore, multiple studies have revealed abnormal DA receptor function in striatal MSNs among human patients and animal models of PD^11–14^, along with structural and functional alterations in MSNs such as dendritic spine loss and changes in the synaptic ultrastructure^15^. Consequently, researchers have speculated that DA-mediated changes in synaptic plasticity within MSNs of the striatum may underlie PD-related motor dysfunction.

Considerable evidence supports the notion that aerobic exercise can significantly ameliorate motor and behavioral impairments in patients with PD. In the realm of animal research, the enforced utilization of the injury-contralateral forelimb in PD model mice has been shown to facilitate the recuperation of motor dysfunction in the affected limb. This approach also mitigates damage to dopaminergic terminals. These beneficial effects are closely linked to exercise-induced plasticity of striatal MSNs, encompassing both morphological and functional aspects. For instance, physical activity facilitates morphological and structural remodeling of striatal MSNs in PD model rats, achieved through the upregulation of synaptic junction protein expression^16^. Concurrently, prior research has demonstrated that physical activity reduces the frequency and amplitude of spontaneous excitatory postsynaptic currents (sEPSCs) and elevates the paired-pulse ratio of D2-MSNs, without altering the fundamental electrophysiological properties of MSNs^17^. Aerobic exercise has also been shown to modulate neuronal excitability by enhancing the expression of D_2_R in striatal MSNs, thereby mitigating motor and behavioral dysfunction in animal models of PD^17^. Nonetheless, the precise roles of the different MSN subtypes in the striatum remain unclear. In the present study, we aimed to investigate the involvement of striatal DA receptors and the associated plasticity of MSNs in regulating motor deficits observed in PD mice during aerobic exercise. In exploring the neurobiological mechanisms mediated by different MSN subtypes, we sought to provide novel theoretical insights into the mechanisms underlying the attenuation of PD-induced deficits in motor function through aerobic exercise.

## Materials and methods

### Ethics information

All experimental methods for this study were carried out in accordance with relevant guidelines and regulations and approved by the Ethics Committee of Hebei Normal University (2020LSC41). All experimental methods were reported in accordance with the ARRIVE guidelines. All efforts were made to minimize the number of animals used and their suffering.

### Animals

Considering the reported male predominance in PD, and in our endeavor to minimize potential experimental confounds that might arise from the variable estrous cycles in female mice, our study design was strategically aligned with these considerations. A total of 54 C57BL/6 male mice (8 weeks old, 20 ± 1 g) were purchased from Changsheng Laboratory Animal Platform (Changsheng, Benxi, China). The C57BL/6 mice were randomly divided into two groups: 6-Hydroxydopamine (6-OHDA) group (n = 32) and saline group (n = 22). The saline group mice were divided into Control group (C, n = 11) and Control + Exercise group (CE, n = 11). A total of 22 successful mice in the 6-OHDA group were randomly divided into PD group (PD, n = 11) and PD + Exercise group (PE, n = 11). The mice had free access to food and water and were housed under a 12/12-h light/dark cycle (natural light) in an animal room with a temperature of approximately 22 °C and relative humidity of 50–60%.

### Adeno-associated virus (AAV) injection

**Figure 1:**
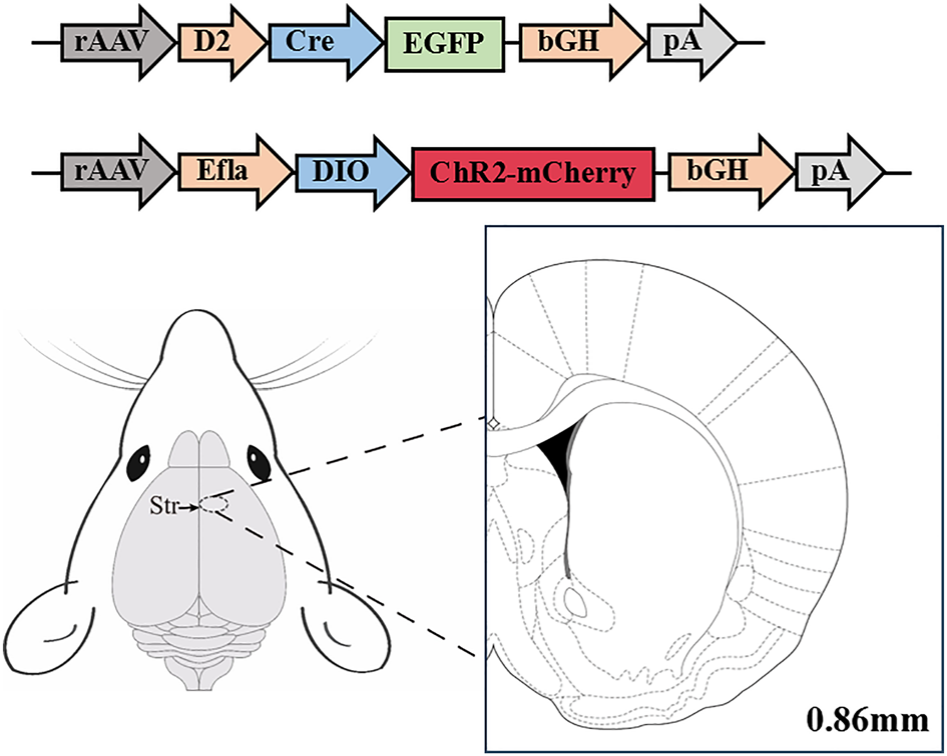
Schematic diagram of Adeno-associated virus injection. The D2 promoter is used to drive Cre expression. When the D2-Cre virus is successfully transfected, ChR2 and mCherry should be driven to be expressed in neurons that express D_2_R.

Mice were anesthetized using 3% isoflurane gas, which was subsequently reduced and maintained at 1%. Deep anesthesia was achieved, and the animals were positioned in a brain stereotaxic apparatus for the surgical procedure. An incision was made in the skin, and the levels of the anterior and posterior fontanelles were adjusted to facilitate localization of the right striatum (AP: + 0.9 mm, ML: − 1.8 mm; DV: − 2.8 mm) based on the mouse brain localization atlas (see Fig. 1). A microinjection pump was utilized to administer a 1 μL mixture of viruses (BrainVTA, Wuhan, China) containing mCherry (2.81E + 12 vg/mL) and D2-Cre (2.37E + 12 vg/mL), with each virus comprising 0.5 μL, at a controlled rate of 100 nL/min. Upon completion of injection, the needle was carefully and uniformly withdrawn.

### Establishment and validation of the PD model

6-OHDA surgery was performed 3 weeks after virus transfection. The mice were deeply anesthetized to align the bregma and lambda of the skull, and posterior holes were drilled at the specified coordinates for the localized striatum (AP: + 0.5 mm, ML: − 1.8 mm; DV: − 3 mm, − 2 mm). A solution of 6-OHDA (Sigma-Aldrich, Saint Louis, USA) with ascorbic acid saline (2 μg/μL) was then injected into the right striatum at a controlled rate of 500 nL/min, at a total volume of 4 μL. In the shamoperated group, an equivalent volume of 0.02% ascorbic acid saline was administered^18^.

On the 7th day post 6-OHDA injection, the PD model mice were assessed using an apomorphine (APO)-induced rotation test. Following intraperitoneal injection of an APO solution (0.5 μg/g), rotations exceeding 120 within a 30-min duration were considered indicative of successful PD model establishment. Mice that passed the apomorphine-induced rotation test were randomly divided into two groups. Mice that failed the rotation test, were excluded from further experiments. The reliability of the PD model was further confirmed via immunohistochemical analysis of tyrosine hydroxylase (TH) expression in the nigrostriatal system.

### Implantation of optical fibers and optoelectrodes

To facilitate data collection during behavioral experiments, an optical fiber insertion needle with a numerical aperture of 0.37 μm and a diameter of 200 μm was utilized. The optical fiber insertion needle was secured on the optical fiber holder, and the position of the striatum (AP: + 0.9 mm, ML: − 1.8 mm) was accurately identified. The needle was then uniformly to a depth of 100 μm below the site of AAV injection.

To facilitate data collection during electrophysiological experiments, a 16-channel optoelectrode was implanted. An opening measuring 1 × 1 mm was drilled above the striatum. The electrode was gradually advanced from this opening at a rate of 0.1 mm/min to approach the dorsal striatum. When this depth was reached and two or more channels of neurons consistently responded to light stimulation, advancement of the optoelectrode was halted.

### Aerobic exercise intervention

According to previous research, aerobic exercise intervention will begin 7 days after 6-OHDA surgery^18^. CE and PE mice underwent a 4-week aerobic exercise intervention using a treadmill designed for small animals, in accordance with the following protocol: treadmill slope of 0°, training speed of 18 m/min, and session duration of 50 min/day. This 50 min duration included a 5 min warm-up and a 5 min cool-down period, during which the running speed was maintained at 10 m/min. The training regimen spanned 4 weeks, with training sessions conducted continuously for 5 days per week. The target exercise intensity was approximately 70–76% of VO_2max_^19^. Before initiating aerobic exercise, exercise intensity was assessed using a metabolic system designed for small animals to ensure compliance with the prescribed standards for aerobic exercise. Maximum oxygen uptake was assessed with the Columbia Small Animal Metabolic System. The testing protocol proceeded as follows: initially, a three-day period of acclimation involving closed gas-exchange treadmill training was conducted before the official assessments. Subsequently, locomotor performance and maximal oxygen uptake were evaluated through a graded incremental methodology applied to treadmill speed for small animals. In the formal assessment, each mouse was positioned on a closed running platform with a 0° incline, utilizing oxygen and carbon dioxide measurements to detect the exhaled gases. Upon stabilization of the animal's oxygen consumption, the running platform initiated at 5 m/min, with speed increments of 4 m/min every 3 min, until the mouse could no longer sustain a specified pace. This threshold was identified as the maximum oxygen uptake and its corresponding running speed. Utilizing the running speed and associated maximal oxygen uptake, the running speed corresponding to 70–76% of VO_2max_ was established for each mouse group.

### Behavioral testing

#### Pole test

To assess motor coordination and impairments, mice were subjected to a pole-climbing test^20^. A climbing pole with a length of 50 cm and a diameter of 1 cm was used, featuring a wooden ball positioned at the top. The mouse was positioned on the ball with its head facing downwards, and the time taken for it to descend from the top to the bottom of the pole was recorded. Each mouse underwent five tests, they were before the exercise intervention and after the weekly exercise intervention. Following completion of the exercise intervention, an optogenetic experiment was conducted in both open and closed optogenetic stimulation states. For the optogenetic stimulation stage, the light was on throughout the entire duration of the trial. Blue light stimulation with a wavelength of 473 nm, power ranging from to 1–5 mW, frequency of 10 Hz, and a pulse width of 20 ms was employed.

#### Cylinder test

The cylinder test is primarily used to assess forelimb asymmetry in hemiparkinsonian animals. Generally, when placed in a cylinder, a mouse explores and adapts to its surroundings by moving and using its forelimbs to touch the walls. In the hemiparkinsonian model, impaired control of left forelimb movement results in an asymmetry in bilateral limb usage. During the experiment, the mice were placed in a cylinder measuring 15 cm in height and 12 cm in diameter, and contact of the forelimbs with the cylinder wall was recorded for 3 min. Left forelimb usage was calculated as follows: (number of left paw contacts + number of bilateral paw contacts/2)/(number of left paw contacts + number of bilateral paw contacts + number of right paw contacts) × 100%^21^. The optogenetic stimulation mode used for the cylinder test matched that used for the previous behavioral test, and the intensity, frequency, and wavelength were in accordance with the plan outlined by Yoon^22,23^.

### In vivo electrophysiological recording

**Figure 2:**
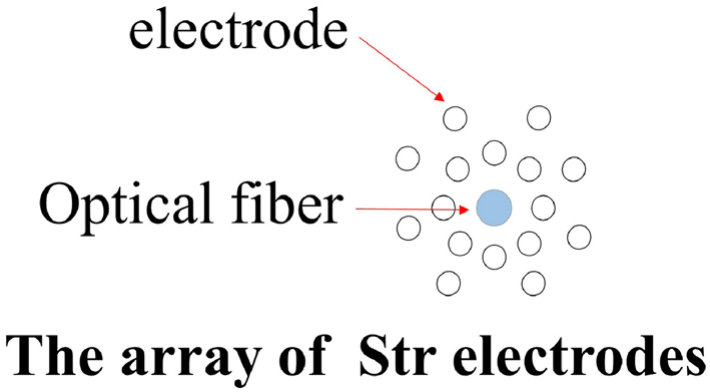
Schematic diagram of photoelectric pole array. From the bottom to top view of the optoelectric electrode, a total of 16 recording electrodes are arranged around the optical fiber.

Following completion of the exercise intervention, a 128-channel neural electrophysiological recording system was used to collect, amplify, and filter electrical signals in the striatum. To activate the D2-MSNs, an intelligent dual-light source optogenetic system utilizing 473 nm blue light was employed (Fig. 2). The recorded signals were extracted through a headstage, and the extracellular action potentials of striatal neurons (Spikes, sampling rate of 30 kHz) and LFP (bandpass filter set to 0.5–250 Hz, sampling rate of 2 kHz) were recorded separately. Electrical signals were collected during baseline and optogenetic stimulation states for each mouse group. As delineated by Wei et al. (2022), an effective signal-to-noise ratio exceeding 3:1, coupled with an automated waveform detection function, establishes thresholds that segregate individual channel waveforms into distinct clusters. This segregation is accomplished through the utilization of Principal Component Analysis (PCA). Subsequently, the analysis involves examining whether the histogram of peak-to-peak intervals adheres to a Poisson distribution (see Fig. 6C). Additionally, this process entails the extraction of both the peak-to-valley and peak-to-peak durations from the characteristic curves of each neuronal waveform (refer to Fig. 6D–F). Refer to Soares-Cunha et al. to distinguish D1-MSNs from D2-MSNs^24^. The parameters for optogenetic stimulation mode were 473 nm of blue light with a power range of 1–5 mW, frequency of 10 Hz, and pulse width of 20 ms.

### Molecular experiments

#### Western blotting

Mice were deeply anaesthetised using isoflurane and then euthanised by decapitation. Mice brains were immediately removed by dissection. The striatum tissues were harvested and protein samples were prepared by tissue lysis. For Western blotting analysis, 20 μg of the striatum protein sample was loaded onto an SDS-PAGE gel and separated using constant voltage mode electrophoresis. Subsequently, the proteins in the gel were transferred onto preactivated polyvinylidene fluoride (PVDF) membranes. To prevent nonspecific binding, the membranes were blocked with 5% skim milk in TBST for 2 h. Following the blocking step, the membranes were incubated overnight at 4 °C with primary antibodies targeting the specific protein of interest (TH, 1:1000, HUABIO; D_1_R/D_2_R, 1:1000, Affinity) as well as β-actin and GAPDH (1:10,000, ABclonal) as a loading control. After incubation with the primary antibodies, the PVDF membranes were washed with TBST and incubated with a secondary antibody (HRP goat anti-rabbit IgG, 1:10,000, ABclonal) that specifically binds to the primary antibody at room temperature for 1 h on a shaker. The chemiluminescent signal was visualized using the FusionFX automatic imaging system.

#### Immunohistochemistry

The mouse brains were swiftly removed and fixed in 4% paraformaldehyde for 24 h. After fixation, the brain tissue was dehydrated using a sucrose gradient solution (20%, 30%, and 30% in PB) and embedded in the OCT compound. The embedded tissue was frozen on a cryostat, and coronal sections (thickness: 15 μm) were obtained once the target brain region had been reached. To retrieve antigens, the sections were initially treated with an antigen retrieval buffer (pH = 6.0). Subsequently, brain slices were incubated with an endogenous peroxidase inhibitor (PV-9001, ZSGB-BIO) at room temperature for 20 min. Primary antibody (TH, 1:200, HUABIO) was then added to the sections and incubated for 1 h at 37 °C. A reaction enhancer was applied to cover the tissue, which was incubated at room temperature for 20 min. Next, HRP-conjugated goat anti-rabbit IgG was added, and the sample was incubated for an additional 20 min, following which DAB staining was performed. In addition Brain sections were washed with 0.01 M PBS between the above steps. The brain sections were dehydrated using different concentrations of ethanol and xylene and were finally sealed with a neutral resin adhesive to preserve the tissue.

#### Immunofluorescence

Brain slices were thawed from − 80 °C and allowed to reach room temperature. They were then washed three times for 5 min each using a 0.01 M PBS solution. Following washing, brain slices were placed in a buffered repair solution for antigen retrieval. The buffered repair solution was boiled in a microwave oven for 6 min in high mode and then for 10 min in low mode. Subsequently, the brain slices were removed and allowed to cool to room temperature. They were washed three times for 5 min each with PBS. For incubation with the primary antibody solution, the sections were carefully treated with a diluted solution containing BSA and the primary antibody (D_2_R, 1:500, Affinity) at 4 °C overnight. The next day, the slices were brought back to room temperature and incubated with the secondary antibody (1:500, Immunoway) for 50 min at 37 °C. Subsequently, the cells were washed three times with PBS for 5 min each. Finally, sections were incubated with DAPI (Cell Signaling Technology, Boston, USA) and sealed with an antifluorescence attenuator. Panoramic images were obtained using a panoramic electronic display microscope. Fluorescent colors represent DAPI (blue) and D_2_R (red).

### Statistical analysis

The data were analyzed using SPSS 25.0 and GraphPad Prism 8 software. Repeated-measures analyses of variance (ANOVA) were used to compare behavioral indices before and after aerobic training in different groups of mice. The effects of the three main factors (aerobic training, 6-OHDA, and training time) on motor behavior were evaluated in each group of mice. When interactions between any of the three main factors was statistically significant (*P* < 0.05), further analysis was conducted to examine the main effects at different levels of aerobic training and 6-OHDA. Conversely, when there were no significant interactions between factors (*P* > 0.05), the main effects were compared. When an interaction effect was still present between two factors (*P* < 0.05), a simple effect analysis was performed, and a two-way analysis of variance was employed to compare variables after aerobic training. The main effects of aerobic training and 6-OHDA were assessed, and if the interaction effect was not significant (*P* > 0.05), the main effects were compared. However, if the interaction effect was significant (*P* < 0.05), a simple effect analysis was performed, and the paired-samples t-test was used to compare behavioral indicators within each group before and after light stimulation. Multiple comparisons were conducted using the least-significant-difference (LSD) test, and the level of statistical significance was set to *P* < 0.05.

## Results

### Evaluation of PD model mice

**Figure 3:**
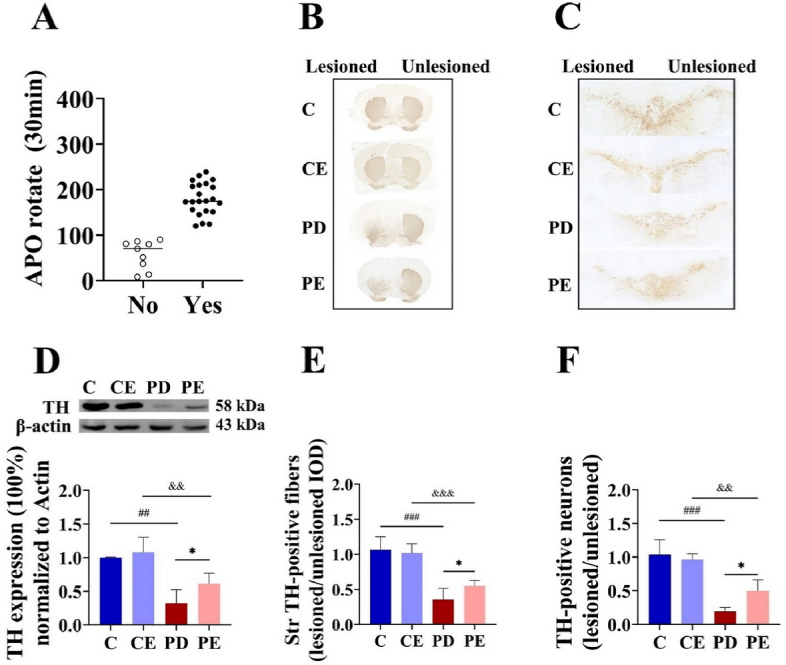
Evaluation results of PD mouse model (**A**) Number of APO-induced rota-tions in mice within 30 min; (**B**,**C**) Immunohistochemical staining of TH in the striatum and SNpc of each mouse group; (**D**) TH protein expression in the right striatum (n = 5); (**E**) Comparison of TH-positive nerve fibers expression in the right/left striatum between mouse groups (n = 6); (**F**) Comparison of TH-positive cells in the right/left SNpc (n = 4). For statistical significance comparisons, the following symbols were used: ^##^C vs PD, *P* < 0.01; ^###^C vs PD, *P* < 0.001; *PD vs PE, *P* < 0.05; ^&&^CE vs PE, *P* < 0.01.

As shown in Fig. 3A, 22 mice exhibited rotational cycles exceeding 120 turns/30 min. Hence, the final success rate for establishing a lateral PD model in this study was 68.75%. After randomized grouping, there was no statistically significant difference in the number of rotational turns between PD and PE mice (Supplementary Fig. S1). Figure 3D shows the western blotting results for TH protein expression in the striatum in each mouse group. Striatal TH loss proportion were significantly higher in the PD group than in the control group (1.00 ± 0.01 vs 0.32 ± 0.20, *P* < 0.01, F = 53.322). PE mice exhibited significantly lower loss proportion of striatal TH than the PD group (0.32 ± 0.20 vs 0.61 ± 0.16, *P* < 0.05, F = 10.032). Furthermore, TH protein expression in the striatum was significantly lower in PE mice than in CE mice (0.61 ± 0.16 vs 1.08 ± 0.21, *P* < 0.01, F = 28.168).

As shown in Fig. 3, integrated optical density (IOD) values for TH-positive nerve fibers and numbers of TH-positive neurons in the SNpc were significantly lower in the PD group than in the control group (0.99 ± 0.16 vs 0.34 ± 0.43, *P* < 0.01, F = 73.015; 1.04 ± 0.22 vs 0.19 ± 0.07, *P* < 0.001, F = 65.333). In contrast, both values were significantly higher in the PE group than in the PD group (IOD: 0.34 ± 0.43 vs 0.57 ± 0.82, F = 7.367; cell number: 0.19 ± 0.07 vs 0.50 ± 0.16, *P* < 0.05, F = 8.061).

### Evaluation of optogenetic model

To achieve targeted expression of red fluorescence in transfected striatal neurons, the rAAV-EF1α-DIO-hChR2(H134R)-mCherry-WRPE-hGH polyA virus was administered. This virus enables visualization of mCherry red fluorescence. As depicted in Fig. 5A, significant red fluorescence was detected in the right striatum of virus-injected mice, whereas no fluorescence was observed in the left striatum, indicating successful viral transfection exclusively on the right side of the striatum.

### Effects of aerobic training and optogenetic stimulation on motor function in each mouse group

**Figure 4:**
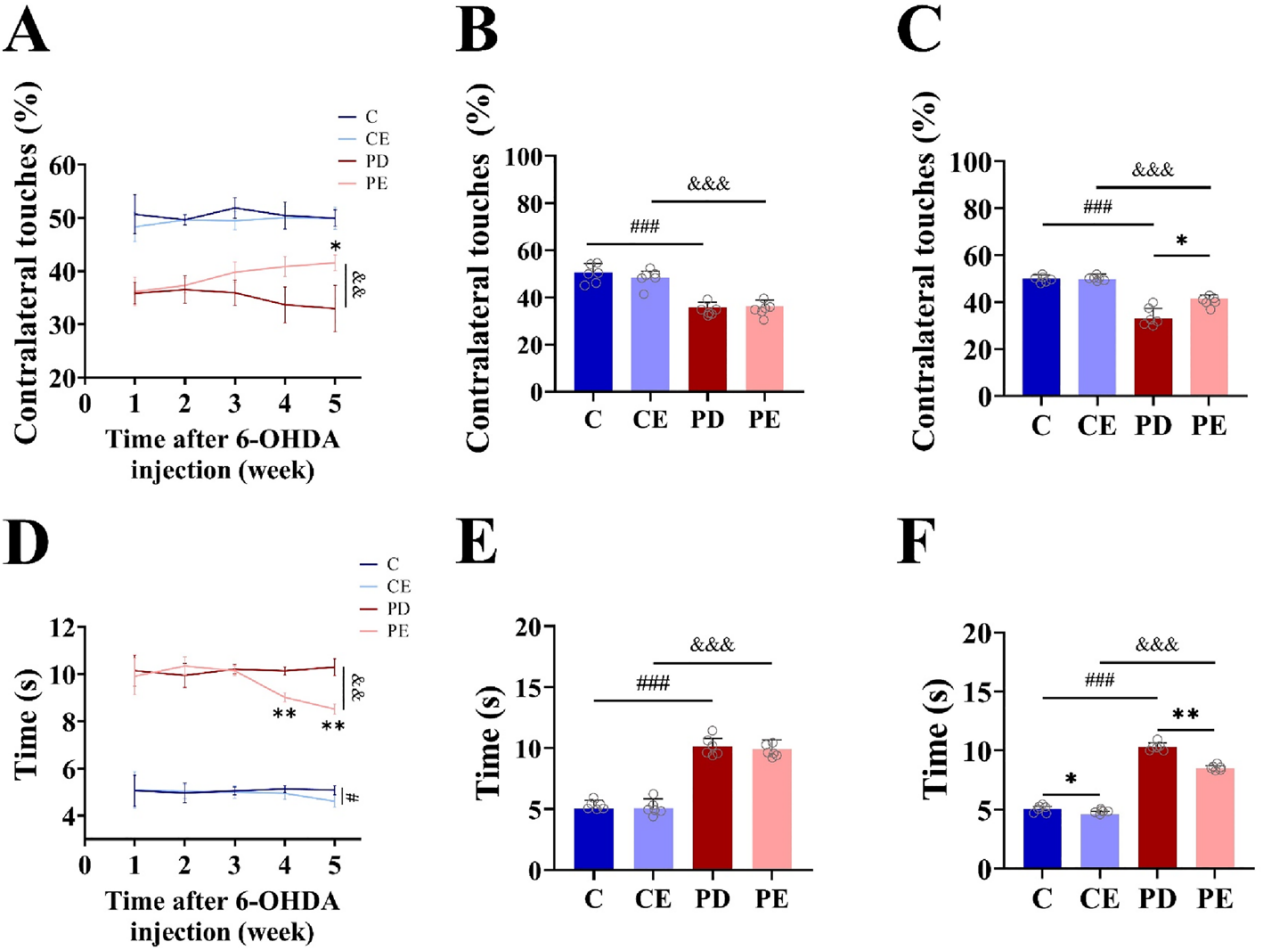
Effects of 4 weeks aerobic training on exercise behavior in mice (**A**) The impact of 4-week aerobic training on the rate of left forelimb usage in each group of mice; (**B**,**C**) The forelimb usage comparisons among the different groups of mice before and after aerobic training; (**D**) The effect of 4-week aerobic training on pole test time in each group of mice; (**E**,**F**) The pole test time comparisons among the different groups of mice before and after aerobic training (n = 6). PD vs PE; ^&&^*P* < 0.01. PE pre-training vs 4-week aerobic training, *P < 0.05; PE pre-training vs 3-week aerobic training, **P < 0.01. Additionally, C vs. CE, ^#^*P* < 0.05.

Figure 4A illustrates the results of the cylinder test, indicating a significant increase in the use of the left forelimb by mice in the PE group after 4 weeks of aerobic training (36.17 ± 2.67 vs 41.58 ± 1.47, *P* < 0.05, F = 18.869). Prior to aerobic exercise, the utilization frequency of the left limb in the PD mouse cohort was markedly reduced relative to the control group. (Fig. 4B, 50.67 ± 3.68 vs 35.82 ± 2.04, *P* < 0.001, F = 54.569). When compared with the PD group, the PE group exhibited a significant increase in the use of the left forelimb after the third week of aerobic training (Fig. 4C, 33.68 ± 3.41 vs 40.86 ± 1.85, *P* < 0.01, F = 20.488). In the pole-climbing test (Fig. 4D), the time taken by mice in the PE group to climb the pole decreased significantly from the pre-test baseline after 4 weeks of aerobic training (9.91 ± 0.77 vs 8.51 ± 0.21, *P* < 0.01, F = 18.084). Additionally, before the onset of aerobic exercise, the climbing duration for the PD mouse cohort was significantly prolonged in comparison to the control group (Fig. 4E, 5.06 ± 0.66 vs 10.15 ± 0.66, *P* < 0.001, F = 150.105). Moreover, when compared with the PD group, the PE group demonstrated a significant decrease in the time required to climb the pole after 3 weeks of aerobic training (Fig. 4F, 10.14 ± 0.17vs 9.01 ± 0.20, *P* < 0.01, F = 110.845).

**Figure 5:**
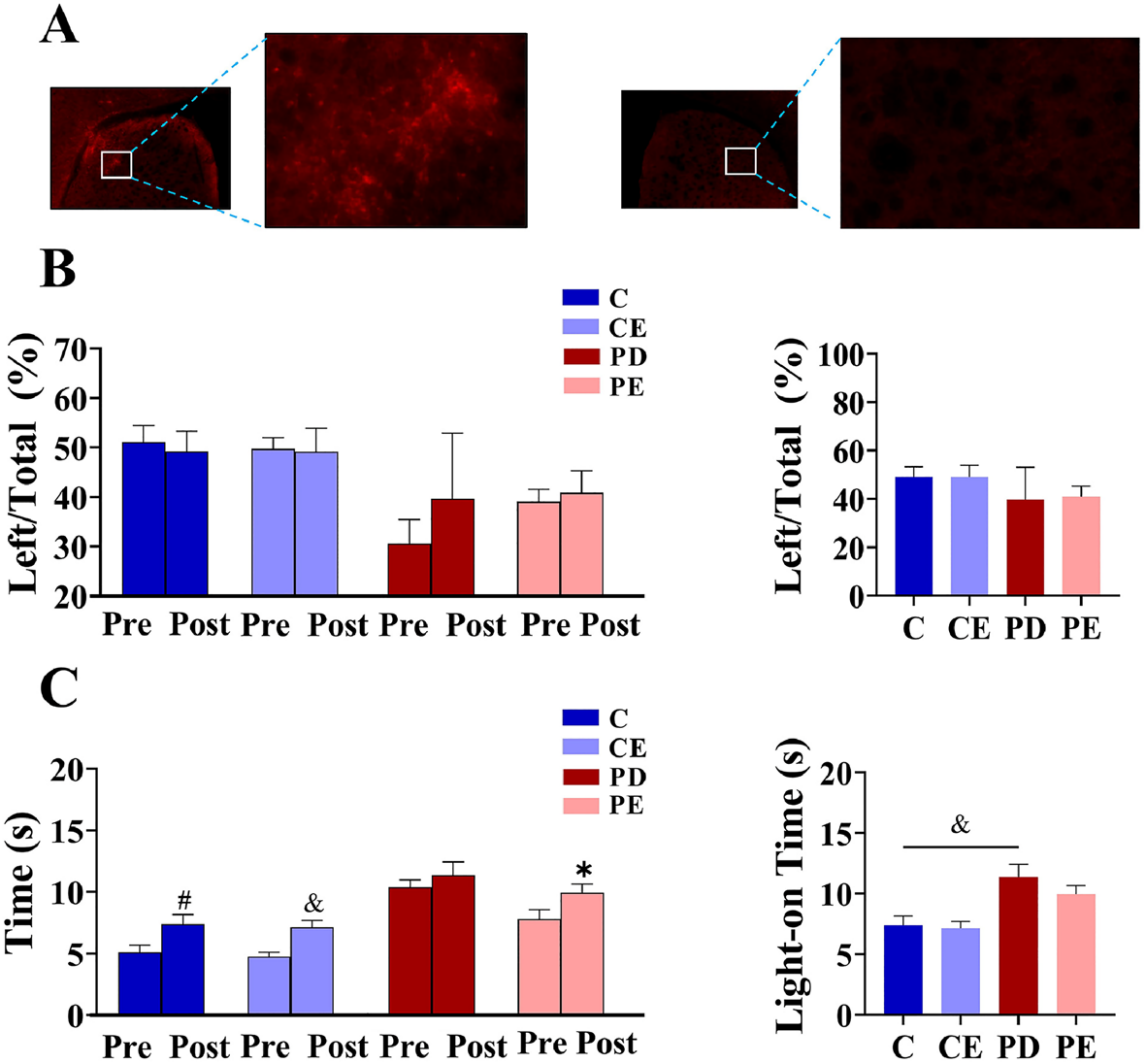
Effect of optogenetic stimulation on motor behavior of mice and evaluation results of mouse optogenetic model. (**A**) expression of dorsal striatal AAV protein on the right and left side; (**B**) Changes in left forelimb usage rate before and after light stimulation, the left figure shows the intra group comparison of each group before and after light stimulation, left figure shows the comparison results between groups; (**C**) Changes in the time of pole test before and after light stimulation (n = 6).

As shown in Fig. 5B, in the cylinder test, there was no significant change in the rate at which the left forelimb was used before and after optogenetic stimulation in any group (*P* > 0.05). As shown in Fig. 5C, climbing time in the pole test was significantly higher in the C, CE, and PE groups after optogenetic stimulation than before stimulation (51.12 ± 3.35 vs 49.21 ± 4.02; 49.74 ± 2.23 vs 49.20 ± 4.69; 39.16 ± 2.48 vs 40.95 ± 4.47, *P* < 0.05, F = 34.407, 76.813, 25.423), while there was no significant change in the pole-climbing time in PD mice before and after photostimulation (*P* > 0.05). The pole-climbing time was significantly higher in the PD group than in the control group after photostimulation (11.37 ± 1.08 vs 7.40 ± 0.77, *P* < 0.05, F = 53.580).

### In vivo electrophysiological results

#### Dorsal striatal neuron fractionation

**Figure 6:**
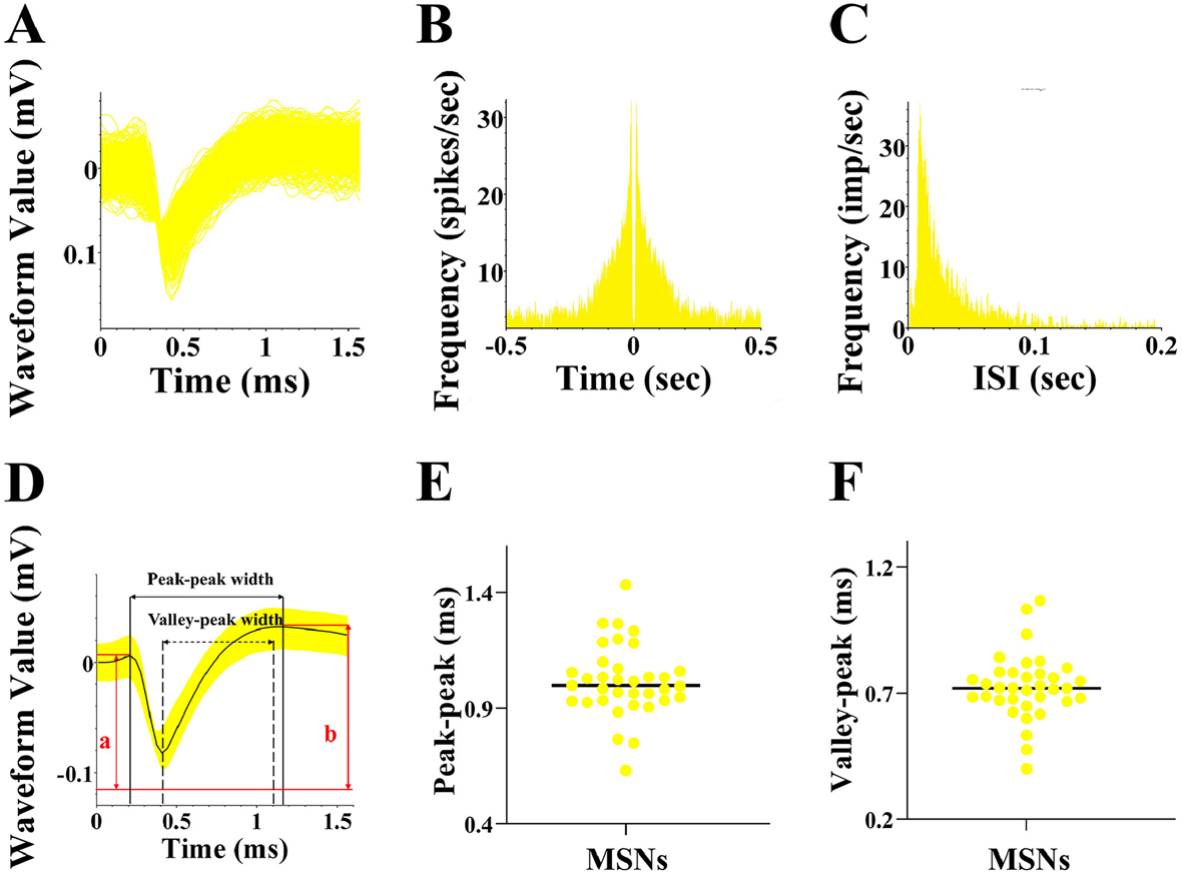
Striatum MSNs waveform features. (**A**,**D**) Summary and characterization plots of dorsal striatal MSNs waveforms; (**B**,**C**) Autocorrelation plots of dorsal striatal MSNs release and histogram of release interval; (**E**,**F**) Statistical plots of dorsal striatal MSNs peak–trough interval and peak–peak interval.

Based on neuronal classification waveform and frequency criteria (Fig. 6), 35 MSNs exhibiting distinct distributions across the three main components with well-defined baseline firing frequencies were identified^25^. The MSNs were distinguished based on extracted waveform features such as spike waveform valley interval, peak interval, and waveform symmetry index.

**Figure 7:**
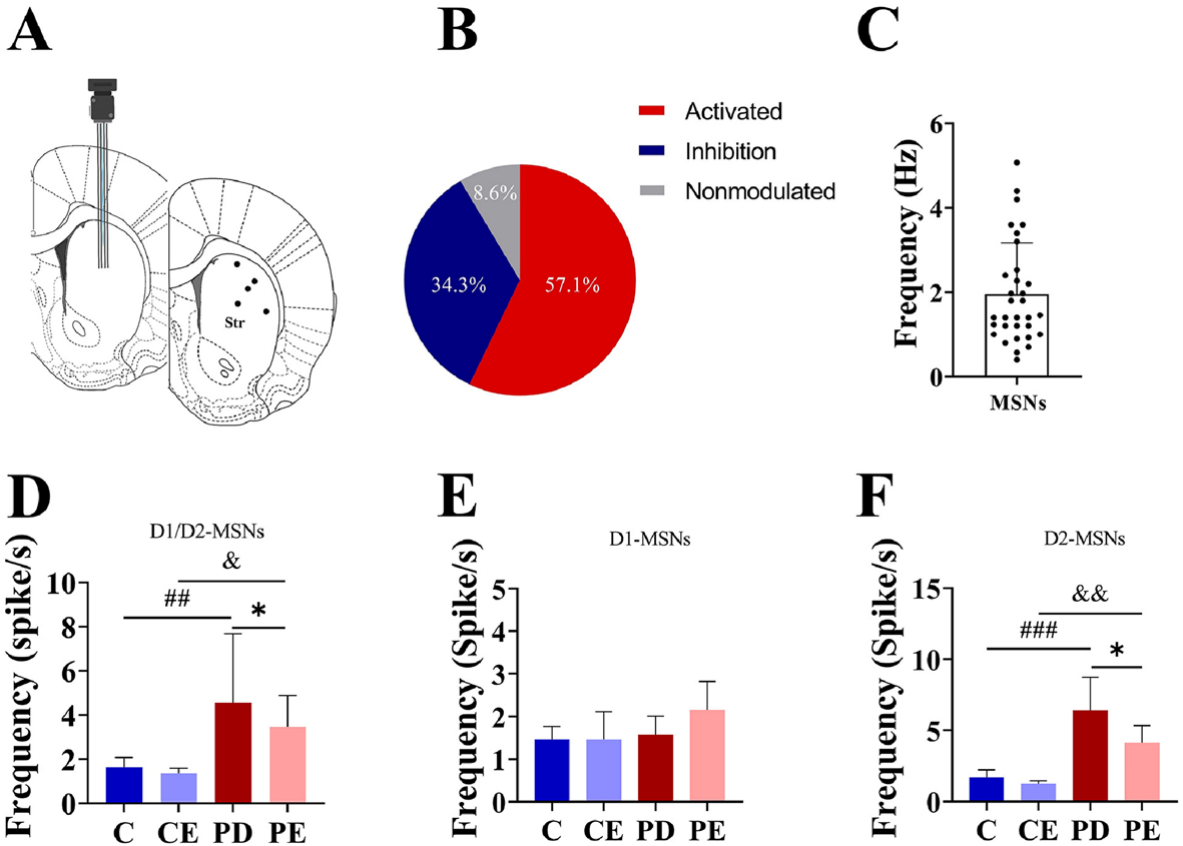
MSNs frequency analysis of dorsal striatum. (**A**) Schematic diagram of electrode implantation sites; (**B**) Statistics of response to light stimulation of dorsal striatal neurons; (**C**) Frequency of neuronal discharges (Hz); (**D**) Statistical graph of overall firing frequency of D1-MSNs and D2-MSNs in the dorsal striatum (Spike/s) (n = 9); (**E**) Statistical graph of firing frequency of D1-MSNs in the dorsal striatum (n = 3); (**F**) Statistical graph of discharge frequency of dorsal striatum D2-MSNs (n = 6). ^##^C vs PD, *P* < 0.01; ^&^CE vs PE, *P* < 0.05; *PD vs PE, *P* < 0.05; ^##^C vs PD, *P* < 0.001; ^&&^CE vs PE, *P* < 0.01.

Figure 7B demonstrates the discrimination of striatal D1-MSNs and D2-MSNs using light activation. Among the 32 neurons responsive to blue light activation at 473 nm, 12 neurons were identified as putative D1-MSNs, accounting for 34.3%, while 20 neurons were identified as putative D2-MSNs, accounting for 57.1%.

### Effects of aerobic training on different MSNs in PD mice

**Figure 8:**
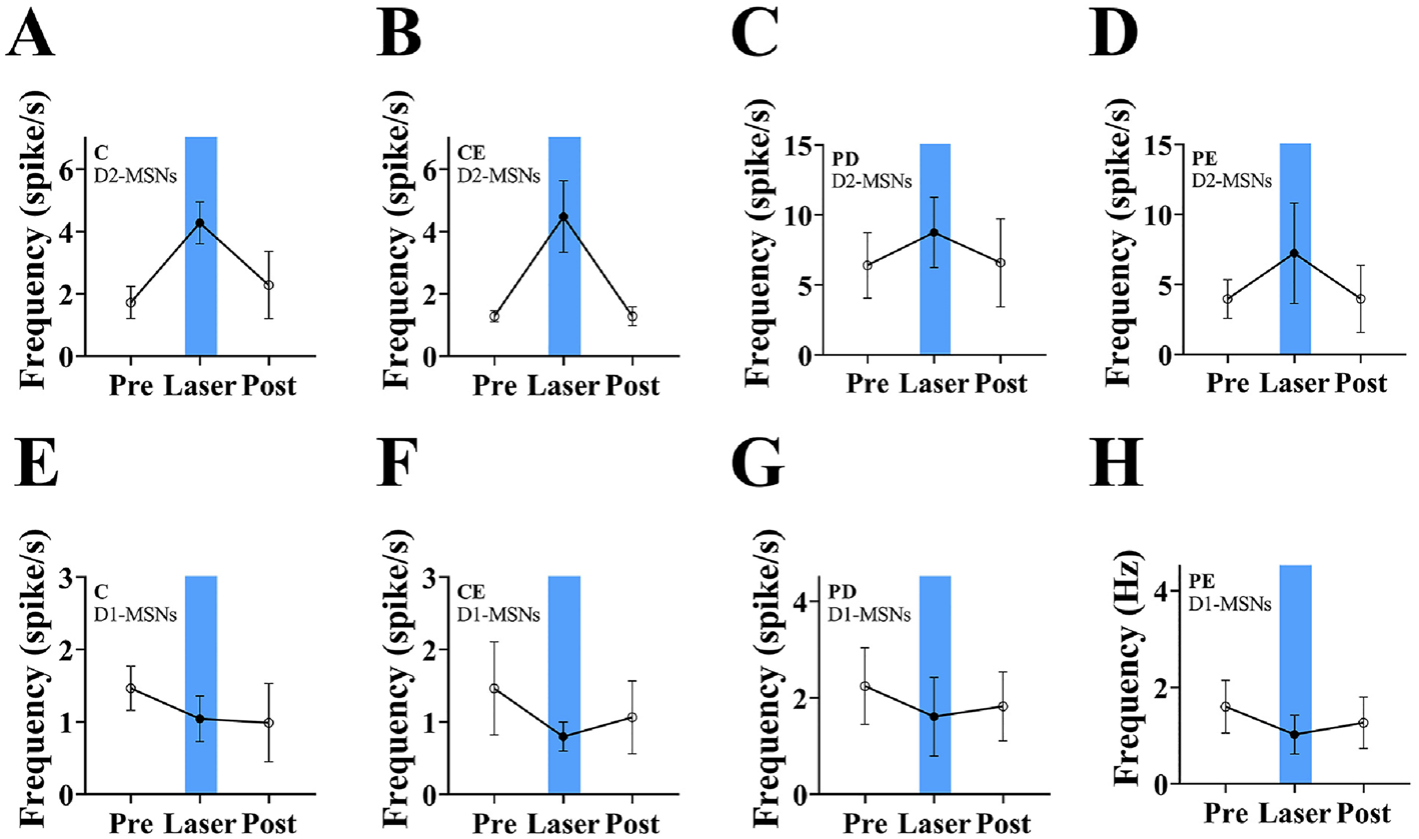
Changes in striatal MSNs response in each group of mice after light. (**A**–**D**) Changes of D2-MSNs frequency in each group (n = 6); (**E**–**H**) Changes of D1-MSNs fre-quency in each group (n = 3).

Electrode implantation sites were all dorsal striatum (Fig. 7A). According to the method of Soares-Cunha et al., neuronal activation was determined by the degree of response of different neurons to optogenetic stimulation, and neurons were defined as activated by optogenetic stimulation and inhibited by optogenetic stimulation when the 473 nm blue light stimulation was able to make the neurons respond within 5 ms, and when the frequency of the post-stimulation discharges was higher than the baseline value by 20% (D2-MSNs), while it was lower than the baseline value by 20% (Figs. 7B, 8).The baseline discharge frequency of MSNs recorded by the experiment is shown in Fig. 7C. Figure 7D shows the firing frequency of D1-MSNs and D2-MSNs in the dorsal striatum in each mouse group. The overall firing frequency of MSNs was significantly higher in PD mice than in control group mice (1.63 ± 0.44 vs 4.55 ± 3.13, *P* < 0.01, F = 6.869). In contrast, the firing frequency of MSNs in was significantly lower in PE mice than in PD mice (4.55 ± 3.13 vs 3.47 ± 1.41, *P* < 0.05, F = 0.871). Figure 7E shows that there were no significant differences in the firing frequency of D1-MSNs among the groups (P > 0.05). Figure 7F shows that the firing frequency of D2-MSNs in the dorsal striatum was significantly higher in the PD group than in the control group (1.72 ± 0.52 vs 6.40 ± 2.34, *P* < 0.001, F = 19.056), whereas it was significantly lower in the PE group than in the PD group (6.40 ± 2.34 vs 4.28 ± 1.28, *P* < 0.05, F = 4.323).

Figure 8 illustrates changes in the firing frequency of D1-MSNs and D2-MSNs in the dorsal striatum in each mouse group before, during, and after photostimulation at 473 nm. The firing frequency of D2-MSNs increased after light stimulation and decreased at the end of stimulation. Conversely, the firing frequency of D1-MSNs decreased following stimulation. For a more detailed examination of the statistical outcomes, please refer to the supplementary material provided.

### Effect of aerobic training on LFP in PD mice

**Figure 9:**
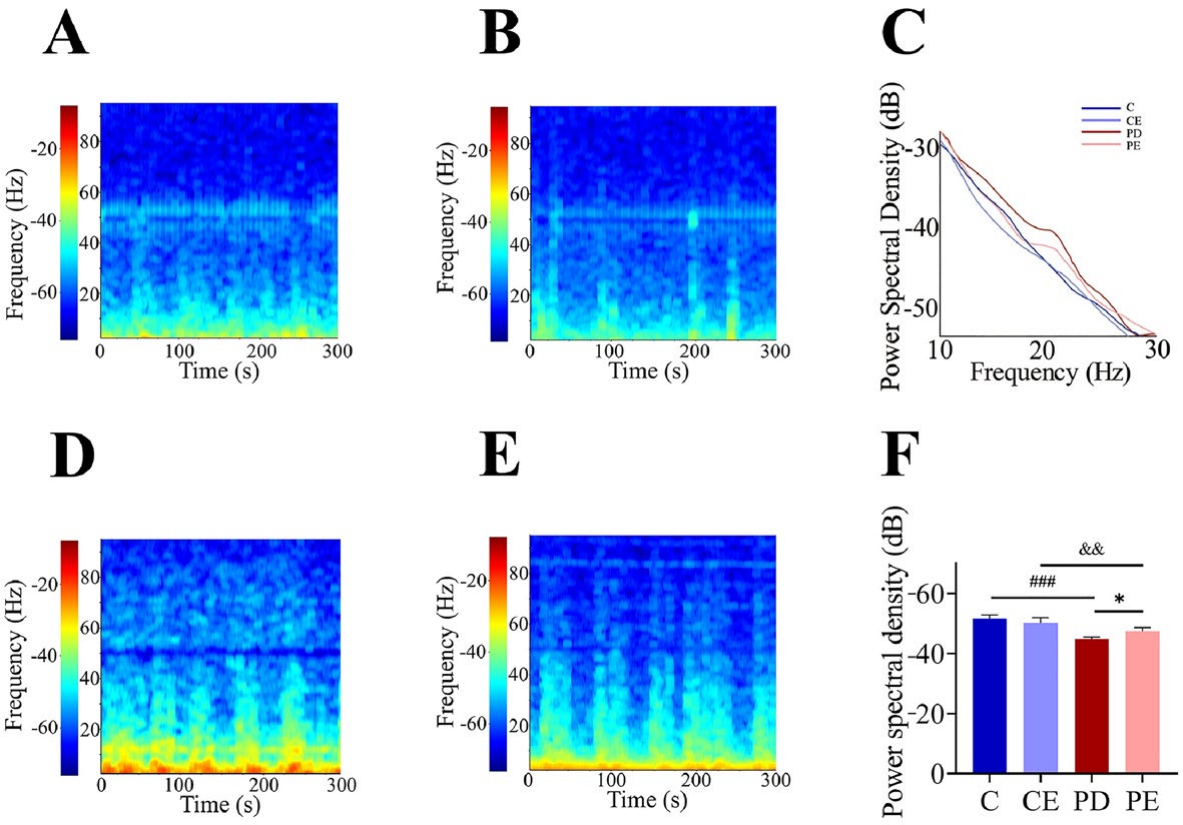
LFP power spectrum of dorsal striatum and β-band power spectrum density. (**A**,**B**) Power spectrum of Group C and CE; (**D**,**E**) Power spectrum of Group PD and PE (n = 4).

As shown in Fig. 9, the power spectral density of LFP in the β-band (12–30 Hz) was significantly higher in the dorsal striatal neurons of PD mice than in those of control group mice (− 51.53 ± 1.35 vs − 44.84 ± 0.70, *P* < 0.001, F = 77.500), while it was significantly lower in the PE group than in the PD group (− 44.84 ± 0.70 vs − 47.39 ± 1.25, *P* < 0.05, F = 12.614).

### Effect of aerobic training on striatal D_1_R and D_2_R in PD mice

**Figure 10:**
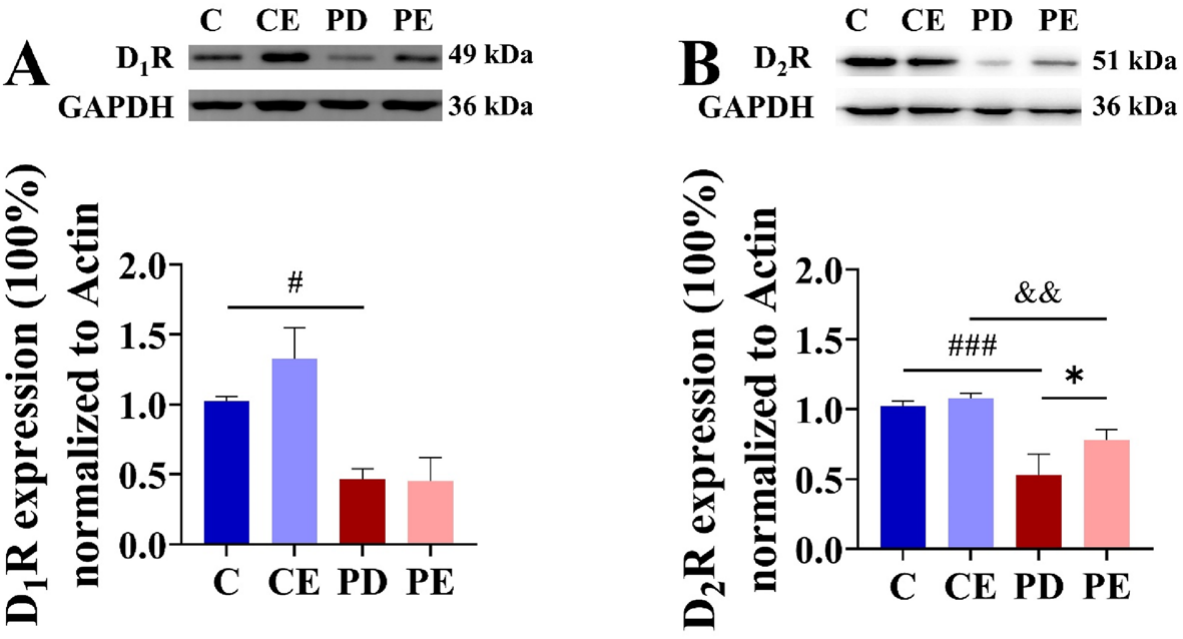
Comparison of striatal D_1_R and D_2_R protein expression. (**A**) Comparison of D_1_R relative protein expression in striatal region (n = 4); (**B**) comparison of D_2_R relative protein expression in striatal region (n = 4). ^#^C vs. PD, *P* < 0.05; ^&^CE vs. PE, *P* < 0.05; ^###^C vs. PD, *P* < 0.001; ^&&^CE vs. PE, *P* < 0.01; *PD vs. PE, *P* < 0.05.

As shown in Fig. 10, levels of D_1_R and D_2_R protein expression in the striatum of were significantly higher in the control group than in the PD group (1.02 ± 0.04 vs 0.47 ± 0.08, *P* < 0.05, F = 13.498; 1.02 ± 0.04 vs 0.53 ± 0.15, *P* < 0.001, F = 40.017). Further, D_2_R protein expression in the striatum was significantly higher in the PE group than in the PD group (0.53 ± 0.15 vs 0.78 ± 0.8, *P* < 0.05, F = 5.136). Notably, there was no significant difference in striatal D_1_R expression between PE and PD mice (0.47 ± 0.08 vs 0.45 ± 0.17, *P* > 0.05).

**Figure 11:**
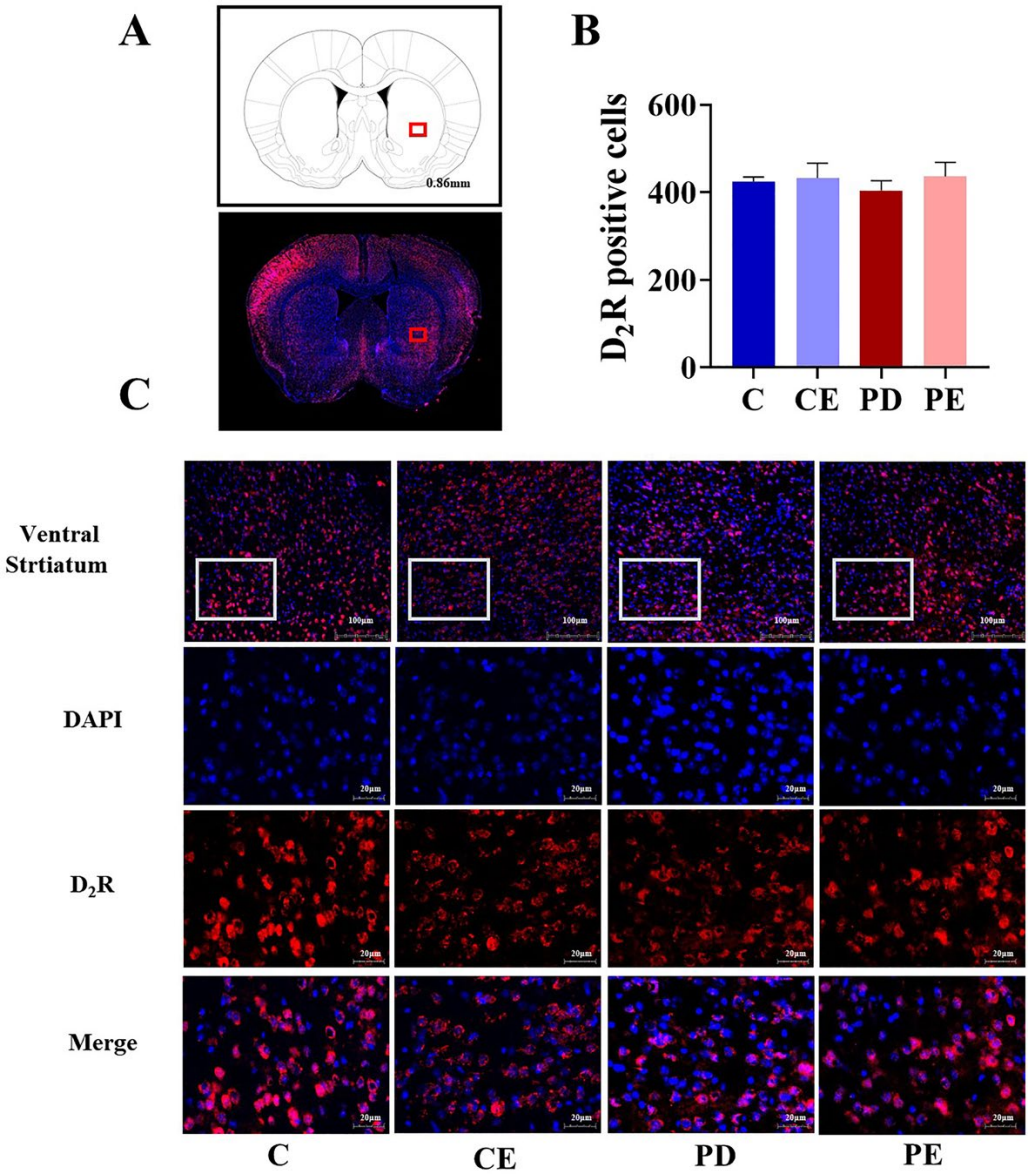
Comparison of D_2_R positive cells expression in the ventral striatal region. (**A**) Schematic diagram of the coronal section of the striatum region in mice, The area where the box is located represents the ventral striatum area; (**B**,**C**) Comparison of D_2_R positive cells in the ventral striatum region of mice in each group (n = 4).

**Figure 12:**
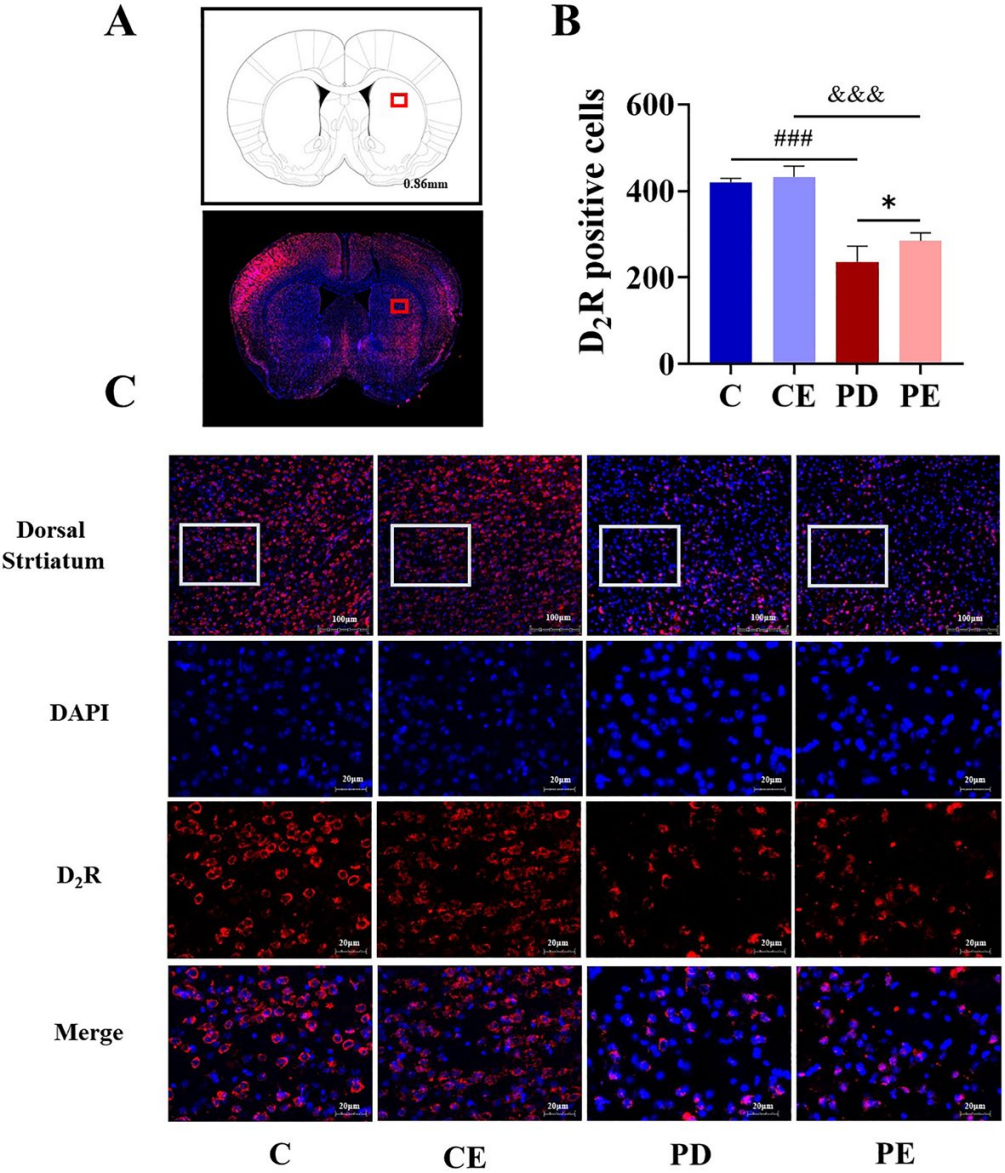
Comparison of D_2_R positive cells expression in the dorsal striatal region. (**A**) Schematic diagram of the coronal section of the striatum region in mice, The area where the box is located represents the dorsal striatum area; (**B**,**C**) Comparison of D_2_R positive cells in the dorsal striatum region of mice in each group (n = 4).

The positive cell number of D_2_R in each brain region was quantitatively analyzed. Figure 11 shows that there were no significant differences in the number of D_2_R positive cells in the ventral striatum among the groups (*P* > 0.05). However, in the dorsal striatum, a significant decrease in the positive cell number of D_2_R was observed in the PD group when compared with levels observed in the control group (419.50 ± 9.68 vs 236.00 ± 36.75, *P* < 0.001, F = 93.253), while a similar, significant increase was observed in PE mice when compared with levels observed in the PD group (284.75 ± 18.69 vs 236.00 ± 36.75, *P* < 0.05, F = 5.591) (Fig. 12).

## Discussion

Epidemiological studies have revealed that engaging in moderate to high-intensity exercise can effectively reduce the risk of developing PD^26^. Accumulating evidence suggests that aerobic exercise not only improves balance, coordination, and aerobic capacity in patients with PD but also attenuates motor symptoms^27^. When compared with resistance and flexibility training, aerobic training has been shown to more effectively enhance functional connectivity between specific areas of the striatum and cortex in patients with PD^28^, thereby improving motor and cognitive function^29–31^. However, the precise roles of and regulatory mechanisms employed by different MSN subtypes in such behavioral improvements remain to be fully elucidated.

Although the degeneration of DA neurons was identified as a key pathological feature of PD in the early 1960s, no specific treatments currently exist to reverse or protect DA neurons. This remains the case despite substantial efforts and resources invested over the past seven decades. However, evidence suggests that Parkinson's disease encompasses a pre-symptomatic phase lasting 10–20 years or longer, typically characterized by the absence of motor symptoms or the presence of only mild symptoms^32^. This scenario presents an opportunity to modify or decelerate the pathological progression from presymptomatic to overt PD. Previous studies have demonstrated that encouraging a biased PD model animal to use its affected limb effectively improves motor deficits in that limb and enhances voluntary activity levels^18^. Such an approach also delays the depletion of striatal tissue DA. However, certain studies indicate that voluntary or forced aerobic exercise only ameliorates motor dysfunction, without mitigating the loss of nigrostriatal DA neurons or enhancing striatal tissue DA levels^33^. This variation could be attributed to the extent of impairment in the PD model relative to the initiation timing of the exercise intervention. For example, in a chronic MPTP-induced mouse model of PD, a 4-week lower-intensity running-stage exercise intervention begun in the third week of modeling failed to alleviate the loss of DA neurons.

In the current study, we observed a significant increase in the time taken by PD mice to climb the pole and a substantial decrease in the utilization of the left forelimb, indicating clear motor dysfunction in these animals. Studies have indicated that more than 50% of DA neurons in the substantia nigra have already degenerated prior to the onset of motor dysfunction in both patients with PD and animal models^34^. Simultaneously, DA levels in the striatum decline in parallel with the loss of DA neurons in the substantia nigra^35^, which affects the regulation of movement by the basal ganglia. Consistent with previous findings, TH levels in substantia nigra/striatum and striatal D_2_R expression were significantly reduced in our PD mice. After 4 weeks of aerobic training, PD mice exhibited significant decreases in climbing time and significant improvements in left forelimb use, suggesting that the aerobic training program was effective in improving motor function in PD mice. Similar outcomes have been reported in other studies^35–37^. Additionally, our study demonstrated that specific activation of striatal D2-MSNs with 473-nm light substantially prolongs the time taken by normal mice to climb the pole and reduces utilization of the left forelimb, highlighting a close relationship between the functional activity of D2-MSNs and motor dysfunction in PD mice. As important components of the indirect pathway within the basal ganglia, striatal D2-MSNs are involved in the initiation, selection, and termination of movement^38^. Optogenetic inhibition of bilateral striatal D2-MSNs disrupts normal movement termination and selection, whereas inhibition of D1-MSNs impairs regular motor behavior^24^. Consequently, improvements in motor dysfunction through aerobic training may be associated with alterations in the functional activity of D2-MSNs.

Previous studies have revealed that the specific spatial organization of dopaminergic and glutamatergic synapses enables DA to influence MSN activity by modulating glutamatergic synaptic transmission^39,40^. The synaptic plasticity of dorsal striatal MSNs, regulated by DA, is closely associated with motor skill learning and motor symptoms in PD^41^. Maintenance of the resting potential in striatal MSNs relies on inward Kir2 K^+^ channels, which modulate the membrane potential by balancing ion flux toward the K^+^ equilibrium potential^7^. Following receptor binding, DA can alter ion channel permeability, thereby influencing neuronal activity. Activation of D_2_R reduces inward depolarizing currents mediated by Nav1 Na + channels and Cav1.l type Ca^2+^ channels while increasing the outward hyperpolarizing K^+^ channel current, thereby facilitating prolonged resting states in D2-MSNs. Conversely, activation of D_1_R via the PKA pathway promotes Cav1 l-type Ca^2+^ channel currents, reduces K^+^ channel currents, and triggers excitation in D1-MSNs^42^. D_1_R and D_2_R colocalize with Glutamate receptors in dendritic spines, representing crucial factors in MSN excitability. Activation of D_2_R reduces Glutamate release from presynaptic terminals and triggers the dephosphorylation of GluR1 subunits, consequently decreasing the AMPAR current on the surface of MSNs. The PKA pathway mediated by D_1_R upregulates AMPAR and NMDAR expression on the membrane surface, thereby regulating ion channel dynamics in the neuronal membrane and affecting neuronal functional activity^43^. Furthermore, D_1_R activation leads to a reduction in NMDA receptor currents and excitotoxicity, while NMDA enhances D1R signaling. Activation of D1 receptors facilitates the upregulation of NMDA-dependent long-term potentiation (LTP). This interaction inhibits the coupling of CaMK-II with NR2B subunits, leading to decreased phosphorylation of NR2B and a reduction in NMDA receptor currents^44–46^.

The functions of postsynaptic neurons are often influenced by the binding of neurotransmitters to their receptor proteins. G protein coupled receptors (GPCRs) play a crucial role in the regulation of inter-neuronal communication by modulating intracellular chemical signals^47^. In the striatum, MSNs express various functional proteins, including D_1_R, D_2_R, adenosine A2A receptors, and glutamate receptors, which are essential for maintaining normal postsynaptic function. D_1_R and D_2_R, both GPCRs, are primarily located on the dendrites of GABAergic MSNs and receive input from dopaminergic neurons in the SNpc and ventral tegmental area, facilitating internuclear communication^48^. Consequently, this leads to decreased inhibition of the thalamus by the basal ganglia and promotes motor behavior. Under normal levels of dopaminergic neurons in the striatum, DA can simultaneously activate both D_1_R and D_2_R receptors. Specifically, DA binds to D_1_R to activate D1-MSNs, while binding to D_2_R inhibits the activity of D2-MSNs. In the absence of DA in the striatum, D2-MSNs lose their inhibition via D_2_R, leading to sustained excitation under the influence of Glutamate^49^. In addition, manipulation of striatal D2-MSNs or D_2_R, either by pharmacological means or optogenetically, affects normal locomotor behaviour in animals^50,51^. Our results similarly show that the presence of motor dysfunction is accompanied by an increased frequency of striatal D2-MSNs issuance and a decrease in D_2_R expression.

Our results indicated a significant decrease in the expression of D_1_R and D_2_R proteins in the striatum, suggesting a close relationship between PD motor dysfunction and DA receptors. Notably, 4 weeks of aerobic training significantly upregulated D_2_R expression in the dorsal striatum of PD mice, while D_1_R levels remained unaffected. Neurotrophic factors, pivotal in mediating neuroplasticity, and improving motor dysfunction in PD through exercise, could contribute to the observed discrepancies. Dating back to 1996, Neeper et al. demonstrated that exercise elevates BDNF mRNA expression across various brain regions in rats^52^. Similar effects have been observed in subsequent studies on exercise interventions in PD^32,53^. Conversely, BDNF-deficient mice exhibit a diminished response to exercise interventions^54^. Furthermore, smilagenin has been shown to safeguard D_2_R and substantia nigra dopaminergic neurons through BDNF in MPTP-induced mouse models^55^. Moreover, BDNF-TrkB signaling in striatal D1-MSNs has a protective effect against the development of levodopa-induced dyskinesias (LID), with targeted activation of TrkB agonists reducing LID severity^56^. Consequently, exercise could mitigate dopaminergic neuron deterioration by activating the BDNF-TrkB signaling pathway, though its protective influence on D1-MSNs may vary from that on D2-MSNs and associated dopaminergic receptors. This suggests that both D_1_R and D_2_R in the striatum play a selective role in ameliorating PD-related motor dysfunction through targeted regulation of D_2_R expression in MSNs in the dorsal striatum. Therefore, 4 weeks of aerobic exercise may improve the excitability of D2-MSNs in PD by selectively modulating D_2_R expression in the dorsal striatum, restoring the normal function of the indirect pathway in the basal ganglia and alleviating PD-related motor dysfunction.

Nevertheless, our study was subject to some primary limitations. Initially, In consideration of variances between species, further investigations are imperative to substantiate the generalizability of the findings from this study to the human population. Secondly, owing to technical constraints, a direct examination of the link between the reduction of striatal DA and the progression of motor dysfunction was not feasible. Therefore, we suggest that the real-time effects of aerobic exercise on brain DA in PD mice should be further monitored in the future.

## Conclusions

Aerobic exercise has shown promising effects in the amelioration of motor dysfunction in patients with PD. Following a 4-week aerobic exercise regimen, we observed a selective reduction in overactivity of D2-MSNs in the dorsal striatum and attenuation of abnormal increases in beta oscillatory electrical activity of LFP in the dorsal striatum. This beneficial outcome was associated with the upregulation of D_2_R expression, which plays a crucial role in improving motor dysfunction in mice with PD. Optogenetic activation of D2-MSNs partially counteracted the effects of aerobic exercise. Notably, our data support the notion that dorsal striatal D2-MSNs have emerged as pivotal cellular targets for enhancing PD-related motor dysfunction through aerobic exercise.

### Supplementary Information

Supplementary Information 1.

Supplementary Information 2.

Supplementary Information 3.

Supplementary Information 4.

## Data Availability

The datasets used and analysed during the current study available from the corresponding author on reasonable request.
