# Supplementary material for: Aerobic exercise improves motor dysfunction in Parkinson's model mice via differential regulation of striatal medium spiny neuron
Source: Sci Rep. 2024 May 27;14:12132. doi: 10.1038/s41598-024-63045-4 (PMC11130133; doi:10.1038/s41598-024-63045-4)
Supplement: Supplementary file 1 — Supplementary Information 1. [file 41598_2024_63045_MOESM1_ESM.docx]

**Statistical Report**

| Figure number | n | Statistic method | | P value | F value | Post hoc multiple comparison test |
| --- | --- | --- | --- | --- | --- | --- |
| Figure 8A | C-D2-MSN  Pre=5  Sti=5  Post=5 | | One-way ANOVA | 0.001 | 14.367 | Pre vs Sti=0.001  Pre vs Post=0.523  Sti vs Post=0.005 |
| Figure 8B | CE-D2-MSN  Pre=5  Sti=5  Post=5 | | One-way ANOVA | 0.001 | 35.165 | Pre vs Sti=0.001  Pre vs Post=0.001  Sti vs Post=1.000 |
| Figure 8C | PD-D2-MSN  Pre=5  Sti=5  Post=5 | | One-way ANOVA | 0.337 | 1.192 | Pre vs Sti=0.377  Pre vs Post=0.993  Sti vs Post=0.434 |
| Figure 8D | PE-D2-MSN  Pre=6  Sti=6  Post=6 | | One-way ANOVA | 0.074 | 3.103 | Pre vs Sti=0.111  Pre vs Post=1.000  Sti vs Post=0.113 |
| Figure 8E | C-D1-MSN  Pre=3  Sti=3  Post=3 | | One-way ANOVA | 0.348 | 1.265 | Pre vs Sti=0.451  Pre vs Post=0.376  Sti vs Post=0.986 |
| Figure 8F | CE-D1-MSN  Pre=3  Sti=3  Post=3 | | One-way ANOVA | 0.310 | 1.434 | Pre vs Sti=0.286  Pre vs Post=0.598  Sti vs Post=0.787 |
| Figure 8G | PD-D1-MSN  Pre=3  Sti=3  Post=3 | | One-way ANOVA | 0.620 | 0.518 | Pre vs Sti=0.603  Pre vs Post=0.792  Sti vs Post=0.940 |
| Figure 8H | PE-D1-MSN  Pre=3  Sti=3  Post=3 | | Paired T-test | 0.415 | 1.022 | Pre vs Sti=0.388  Pre vs Post=0.706  Sti vs Post=0.915 |
