# Supplementary figures and images for: Aerobic exercise improves motor dysfunction in Parkinson's model mice via differential regulation of striatal medium spiny neuron
Source: Sci Rep. 2024 May 27;14:12132. doi: 10.1038/s41598-024-63045-4 (PMC11130133; doi:10.1038/s41598-024-63045-4)

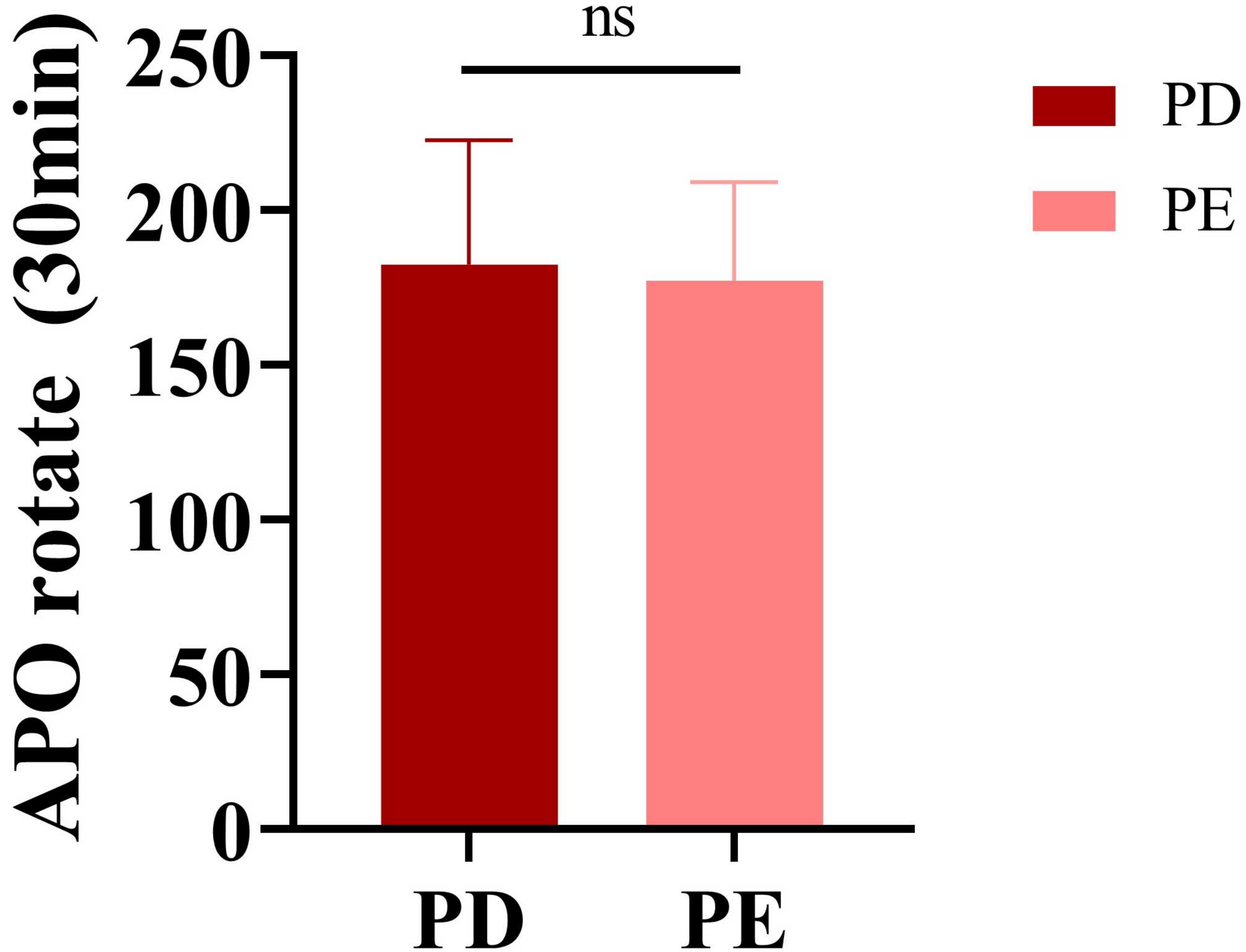

Supplement: Supplementary file 2 — Supplementary Information 2. [file 41598_2024_63045_MOESM2_ESM.pdf]

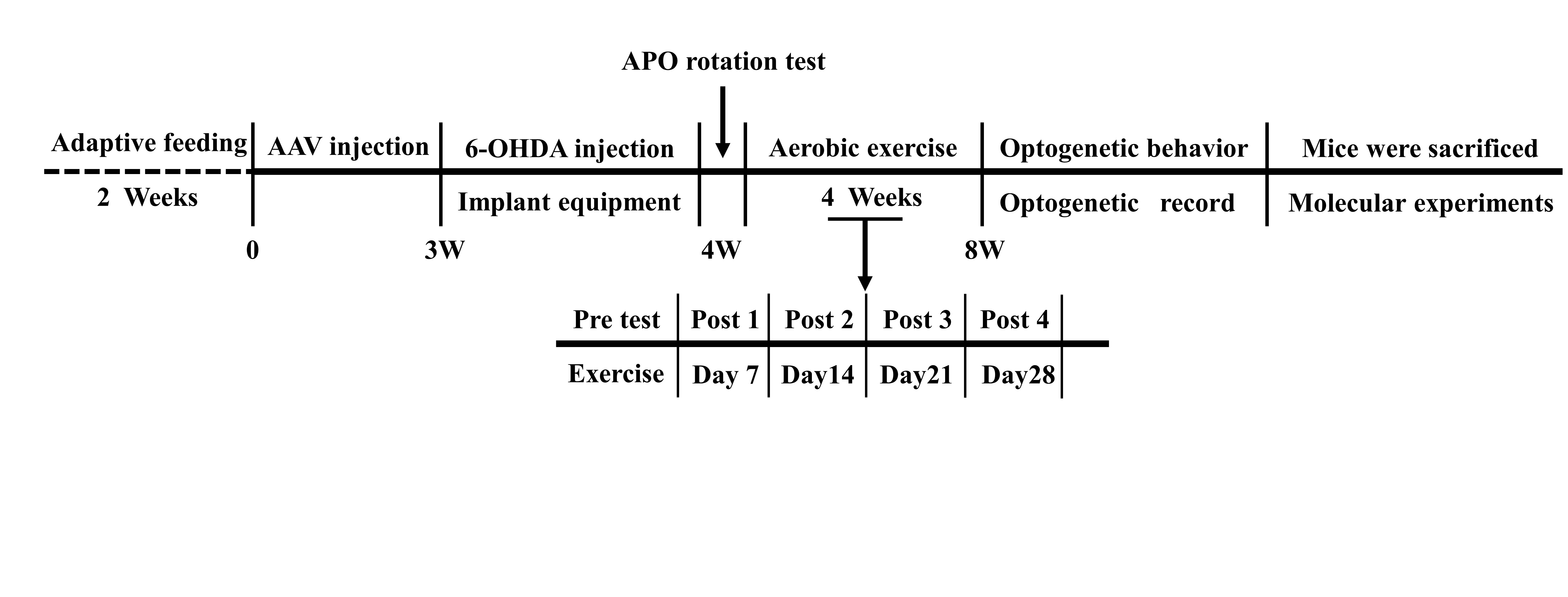

Supplement: Supplementary file 3 — Supplementary Information 3. [file 41598_2024_63045_MOESM3_ESM.jpg]
