# Supplementary material for: Aerobic exercise improves motor dysfunction in Parkinson's model mice via differential regulation of striatal medium spiny neuron
Source: Sci Rep. 2024 May 27;14:12132. doi: 10.1038/s41598-024-63045-4 (PMC11130133; doi:10.1038/s41598-024-63045-4)
Supplement: Supplementary file 4 — Supplementary Information 4. [file 41598_2024_63045_MOESM4_ESM.pdf]

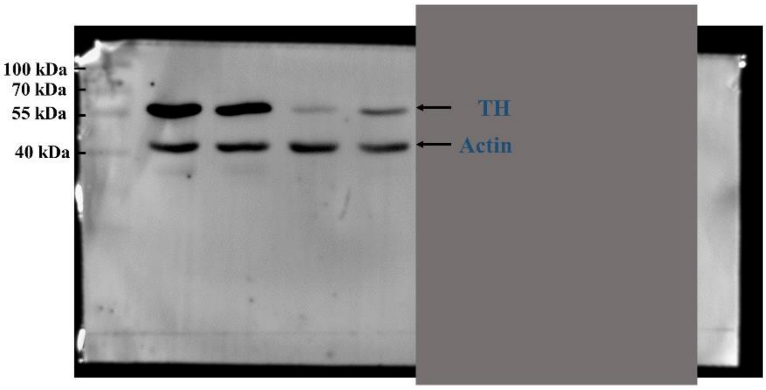

Note: Obscured positions are unpublished experimental data.

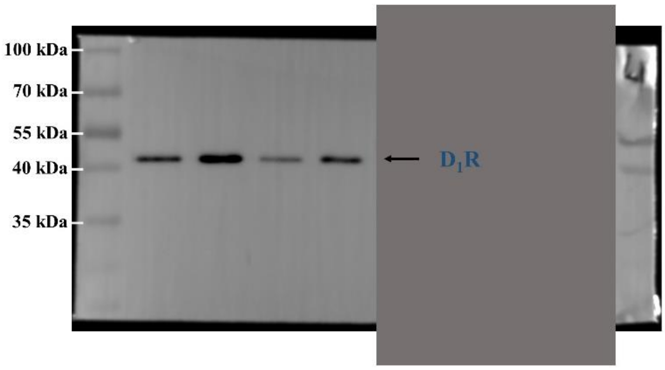

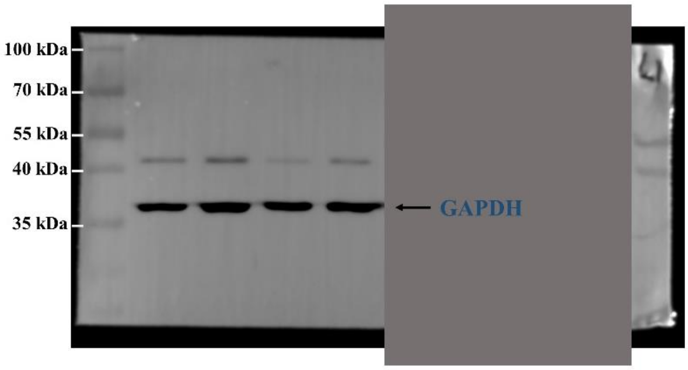

Note: Obscured positions are unpublished experimental data.

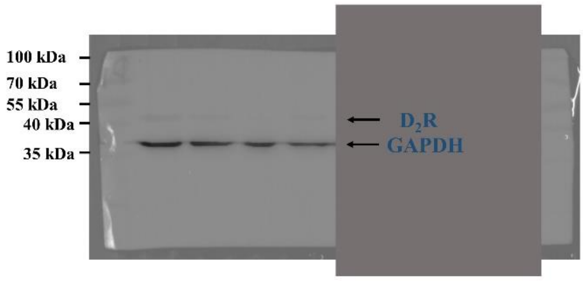

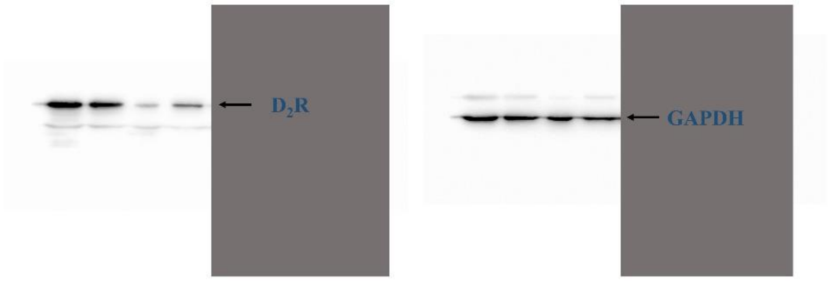

Note: Obscured positions are unpublished experimental data. In addition, the top image shows a schematic representation of the protein locations, and the bottom image shows the protein images used in the manuscript.
